# Creating a community advisory board for pediatric bladder health

**DOI:** 10.3389/fped.2024.1396003

**Published:** 2024-07-16

**Authors:** Emily Teehan, Ashley Phord-Toy, Pranaya Venkatapuram, Kathleen M. Kan

**Affiliations:** School of Medicine, Stanford University, Stanford, CA, United States

**Keywords:** community advisory board, lower urinary tract symptoms, pediatrics, community engagement, urology, community health

## Abstract

**Introduction:**

Pediatric lower urinary tract symptoms (LUTS) are highly prevalent in neurologically healthy school-aged children. However, no evidence-based programs exist to prevent or treat LUTS in the community setting. To address this, we established the first community advisory board (CAB) that aims to identify individual and societal structures impacting pediatric bladder health in Northern California's Bay Area and co-design culturally relevant bladder health interventions.

**Methods:**

Probability and non-probability sampling methods were used to recruit community stakeholders to the CAB. Our final CAB comprised of two parents, two community health workers, one educator, one pediatric urology registered nurse, and one pediatrician. The CAB met quarterly during the 1-year study period.

**Results:**

Bi-directional feedback identified community-level barriers to bladder health, particularly in the school environment, and the need for tailored resources to teach children and families about healthy bladder behaviors.

**Discussion:**

The CAB co-designed school-based bladder health interventions, including bladder health posters, and provided feedback on three school-based research study proposals. The CAB will continue to guide and inform future community-engaged research efforts.

## 1 Introduction

Pediatric lower urinary tract symptoms (LUTS) affect up to 22% of neurologically healthy school children (1). LUTS can be characterized into storage symptoms (i.e., frequency, urgency, daytime incontinence, and enuresis) and voiding symptoms (i.e., hesitancy, straining, weak stream, and intermittency) (2). While early pediatric LUTS may be due to inadequate potty training, wetting symptoms that increase around age 7–8 years of age may be a function of preventable behaviors such as holding of urine and bathroom avoidance (3, 4). Pediatric LUTS can be effectively treated by medical providers via health education and behavioral modification focused on four healthy bladder behaviors: (1) voiding regularly (every 3 hours), (2) adequate hydration and dietary changes, (3) regular bowel movements, and (4) correct toileting behaviors (5). This treatment regimen improves symptoms in most patients, yet only 10%–16% of families with pediatric LUTS report that they will seek medical treatment (6). This statistic highlights a potential gap between available treatment options and delivery of this information to the community members and families who may need it the most.

There is evidence that community-based factors impact the experience of families awaiting medical care and those struggling to adhere to healthy bladder behaviors. Widespread access to pediatric LUTS health information in the community is limited in the absence of public health initiatives to improve pediatric bladder health, and therefore, interventions targeting bladder health are tightly linked to healthcare access. For families who may independently seek information, existing resources on the internet can be outdated and written at a reading level that is too high for adults (7). Bathrooms themselves can be structural factors in the community that contribute to symptoms. Adolescents and adults often report that bathroom environments in schools, workplaces, and public spaces are inadequate due to their lack of cleanliness, leading to increased bathroom avoidance (8–11). Addressing these individual, interpersonal, and community-factors can help promote the adoption of healthy bladder behaviors and improve pediatric LUTS care.

There is a need for bi-directional partnership between healthcare providers and community stakeholders to (1) develop a robust perspective on treatment and prevention of pediatric LUTS in the community setting and (2) identify areas for prioritization in future pediatric LUTS research. Community-engaged (CE) research is a framework that recognizes the strengths of community members to contribute towards improving the effectiveness and impact of policies and programs (12, 13). Community advisory boards (CABs) are a common platform for CE research that provide an infrastructure for a community-academic partnership (14). To our knowledge, a CAB for pediatric bladder health research has not been previously formed. This report presents the theory and results from the development and implementation of the first CAB for pediatric bladder health research aimed at co-creating culturally relevant bladder health interventions for Northern California's Bay Area families. This CAB is a CE research committee that contributes to Kan Lab's CE research infrastructure. The goals of CAB are informed by our previous and ongoing research projects aimed at understanding community-level barriers to pediatric bladder health and adoption of healthy bladder behaviors. Our CAB ensures that our research projects reflect the needs, preferences, and interests of key stakeholders involved in children's bladder health.

## 2 Materials and methods

We aimed to select 6–8 community stakeholders important to the health and learning of children ages 5–10 over a 1-year study period between January 1, 2023 and January 1, 2024. Our goal was to complete four meetings and meet the following deliverables: (1) a CAB Mission Statement, (2) a Letter of Agreement specifying the terms, responsibilities, and compensation for CAB members, (3) an annual report with key decision updates, and (4) the completion of formal community partner training by all research team and CAB members.

### 2.1 CAB member recruitment

**Figure 1:**
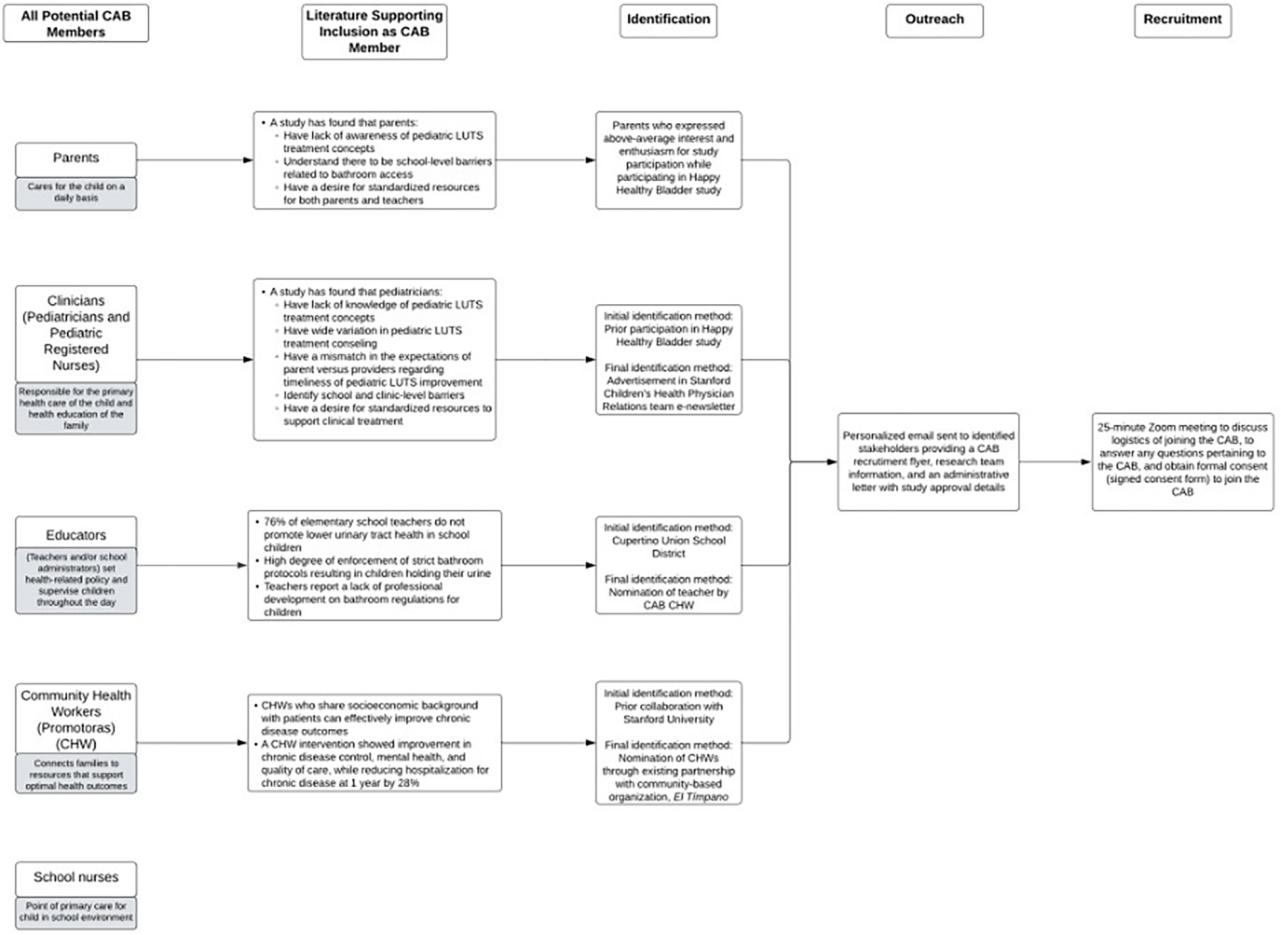
CAB member identification and recruitment flowchart.

Prior to beginning recruitment, a list of all potential community stakeholders was brainstormed based on existing LUTS literature and consultation with our pediatric urology providers to ensure the likelihood of meaningful feedback related to bladder health in the community (Figure 1) (15–25). Recruitment of both English- and Spanish-speaking populations was a priority, as 18% of our pediatric urology practice population speak Spanish and 27% identify as Latinx, Chicanx, or Hispanic (“Hispanic” per AAMC guidelines) (26, 27). Recruitment used both probability and non-probability sampling methods. For example, we recruited through hospital-based e-newsletters disseminated to a network of pediatricians and pediatric nurse practitioners (simple random sampling). Additionally, we leveraged our existing pediatric LUTS research infrastructure and recruited from a pool of parents and pediatricians who had previously completed bladder health studies with our research team (convenience sampling). We also partnered with a Spanish-language community-based organization (CBO), *El Tímpano*, to recruit community health workers (CHWs) with whom they had previously worked (snowball sampling). Successful recruitment was defined as signing of the formal consent document to participate as a member of the CAB.

Once a participant expressed interest, the research team emailed personalized invitations with CAB details to each potential participant. This email contained a CAB recruitment flyer, research team meet n' greet information, and an administrative letter with Institutional Review Board (IRB) study approval details. The participant could then self-schedule a virtual meeting with the research team to review logistics of CAB participation, answer questions, and sign consent for a 1-year study period (four meetings). After consent was obtained, a follow-up email included the signed consent form, answers to remaining questions, and a request to provide meeting availability.

### 2.2 CAB meeting operations

The CAB met quarterly during the 1-year study period on Zoom™ for 90 min. A PowerPoint™ slide presentation was presented on a shared screen. At the conclusion of the first CAB meeting, possible future meeting dates were proposed and feedback guided selection of subsequent meeting dates. Each member received a $199 honorarium split into four payments for attendance and completion of individual deliverables. Prior to each meeting, a general meeting agenda was developed based on the *a priori* defined objectives, ongoing research team projects and CAB member feedback. This was emailed along with meeting reminders prior to each date.

At the beginning of each meeting, a meeting agenda was presented that included time-allotments and mid-meeting breaks to provide clear expectations of how the time would be managed. For meetings #2–4, welcome/agenda slides were followed by a review of takeaways from the previous meeting. This ensured validation of member feedback and confirmed the research team's understanding of findings. This method is consistent with a member checking approach used in qualitative study design (28). Next, we proceeded through the stated agenda topics with (1) group review of training modules and (2) scheduled 5–10 min debrief sessions on open-ended questions aimed at guiding conversation and generating feedback. To guide bi-directional feedback, the Co-Design framework was utilized (29). The Co-Design framework involves six stages: (1) engage and align, (2) explore and connect, (3) imagine and decide, (4) create and test, (5) co-implement and co-evaluate, and (6) share and celebrate (29). We adapted this framework to elicit conversation about bladder health interventions including education resources over the course of all four meetings. Meetings concluded with a plan for the next meeting and a prompt to complete the post-meeting survey. One research team member took detailed minutes, one research team member tracked time and progress through the meeting slides, and all research team members, including the principal investigator, presented different portions of the meeting agenda. After the meeting, feedback was condensed by the research team into “main takeaways”.

If CAB members were unable to attend due to scheduling conflicts, the research team met with the individual(s) at an alternative time to review the same slide presentation, open-ended feedback questions, and training modules. Training could alternatively be completed on an individual's personal time. If a meeting time could not be accommodated before the next meeting, a written Google Form was provided with the same open-ended feedback questions. Feedback from individual meetings and larger CAB meetings was condensed and presented back to the group at the start of meetings #2–4 to ensure all CAB members were aware of the findings.

### 2.3 CAB deliverables

#### 2.3.1 Mission Statement and letter of agreement

During CAB meeting #2, we used a shared decision-making process to design a Mission Statement and outline the responsibilities to be included in the Letter of Agreement. Research team members utilized initial feedback to draft a Mission Statement and Letter of Agreement that were presented during CAB meeting #3. Additional feedback from was incorporated into the final version.

#### 2.3.2 Community partner training

All research team members and CAB members were asked to complete the community partner training program, *Communities Connecting to Research* (30). The goal of the training was to enhance the capacity of CAB members and researchers to enter and collaborate effectively in community-academic partnerships. We selected this curriculum after review of multiple training programs because it covered the topics most relevant to a newly formed CAB, such as the research process, CE research, and vulnerable populations (30). Research team members completed the training prior to the commencement of CAB meetings. CAB members completed the training in CAB meetings #2–4 to minimize work outside of meetings and ensure a high rate of completion.

#### 2.3.3 Annual report

The research team produced a report summarizing CAB operations and thematic findings over the 1-year period. The goal of this document was to present members with a reflection of what the team had accomplished, as well as key decisions regarding ongoing and future research endeavors.

### 2.4 CAB evaluation

To evaluate the CAB from a multi-dimensional perspective, a variety of metrics and data collection methods were identified, as outlined in Supplementary Table S1.

#### 2.4.1 Post-meeting survey

A brief 2-min anonymous survey was developed by the research team to elicit immediate feedback that could improve subsequent meetings. The survey assessed the usefulness of CAB meetings, attitudes towards CAB meeting format and duration, as well as overall preparedness of each CAB member prior to meetings (Supplementary Table S2). A survey link was distributed via the Zoom™ chat feature at the end of the meeting and was included in post-meeting emails.

#### 2.4.2 Adapted clinical and translational science awards (CTSA) CAB implementation survey

An adapted version of the CTSA CAB Implementation survey was delivered at the conclusion of the third meeting on October 23, 2023 (31). The purpose of the survey was to assess member's perceptions and understanding of (1) CAB logistics, (2) CAB's community impact, and (3) challenges experienced during the first year of CAB operations. Survey items were adapted to add the phrase “Kan Lab” to relevant questions. Questions were presented as multiple-choice questions, with numerous questions providing the option to select multiple answers.

**Figure 2:**
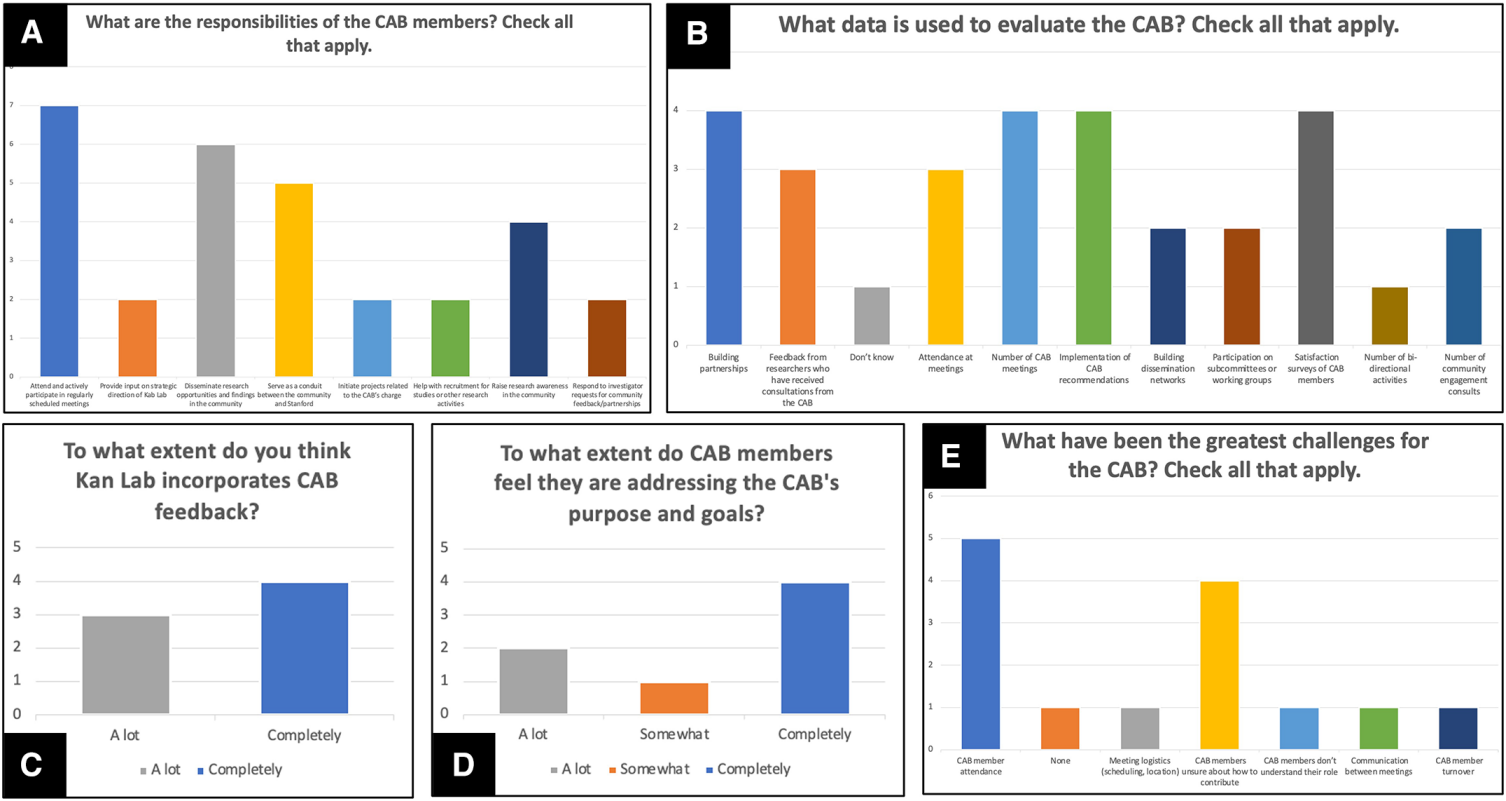
Subset of all results from adapted CTSA CAB implementation survey. These results reflect (**A**) CAB members' perceptions of their responsibilities, (**B**) methods used to evaluate the effectiveness of the CAB, (**C**) Kan Lab incorporation of their feedback, (**D**) the extent that CAB members are addressing the CAB's purpose and goals, and (**E**) the greatest challenges to the CAB. Questions presented in (**A**), (**B**), and (**E**) provided respondents with the option to “check all that apply”. All results are displayed as histograms with the y-axis indicating the frequency of CAB members who selected each answer option on the x-axis.

A subset of all questions included in the adapted CTSA CAB Implementation Survey are presented in Figure 2. These questions assessed CAB members' perceptions of (1) their responsibilities, (2) methods used to evaluate the effectiveness of the CAB, (3) Kan Lab incorporation of their feedback and the extent that CAB members are addressing the CAB's purpose and goals as indicators of community impact, and (4) the greatest challenges to the CAB. To accommodate members who predominantly spoke Spanish but also read and write in English, survey items were translated by our bilingual research team members. The instrument was administered during CAB meeting #3 time to minimize the need for CAB members to use additional personal time, ensure a high completion rate, and allow for live assistance of a bilingual research team member for any comprehension needs. During CAB meeting #4, results were presented, and feedback was requested on areas for CAB improvement.

## 3 Results

### 3.1 CAB members

Recruitment to the CAB began in January 2023 and concluded on August 15, 2023. All appointed CAB members resided in Northern California's Bay Area. Our final CAB was comprised of two parents of pediatric patients with LUTS, two CHWs, one educator, one pediatric urology registered nurse (RN), and one pediatrician. As time progressed, we increased the use of snowball sampling as we asked successfully recruited CAB members to recruit additional members. Recruitment of an educator was not finalized until the conclusion of the first CAB meeting due to scheduling conflicts with an initially recruited educator.

### 3.2 CAB attendance

All CAB members were able to attend the first CAB meeting on June 6, 2023. Attendance for meetings #2–4 ranged from three to six CAB members. Three research team members and the principal investigator attended all CAB meetings. We conducted separate Zoom meetings for CAB meeting #2 (pediatric urology RN) and CAB meeting #3 (pediatrician), and CAB meeting #4 (educator). We administered a Google Form survey for CAB meeting #2 (pediatric urology RN), CAB meeting #3 (parent), and CAB meeting #4 (pediatrician and CHW).

### 3.3 CAB agendas

Due to our initial recruitment period, our four CAB meetings were scheduled in the latter half of a 12-month period, on (1) June 5, 2023, (2) September 6, 2023, (3) October 23, 2023, and (4) December 4, 2023. All meetings were able to cover the stated agenda. The topics, main takeaways, deliverables of each meeting are presented in Supplementary Table S3.

### 3.4 CAB deliverables

#### 3.4.1 CAB Mission Statement

The Mission Statement of the CAB summarizes the over-arching purpose of the CAB and includes three parts: (1) a vision statement, (2) a Mission Statement, and (3) values. The main feedback received was to include prevention of pediatric lower urinary tract symptoms (LUTS) in the Mission Statement, in addition to the treatment of pediatric LUTS.

*Vision Statement:* To improve care for all families in the Bay Area who are experiencing or are at risk of experiencing pediatric lower urinary tract symptoms.
*Mission Statement:*
  • Identify common research goals between researchers and CAB stakeholders.
  • Elicit ongoing feedback for areas of greatest need within the community.
  • Design future solutions that meet community needs effectively by incorporating stakeholder feedback.
  • Empower stakeholders to affect change in future projects.
*Values:* Inclusivity, Empowerment, Quality Care.

#### 3.4.2 Letter of agreement

The Letter of Agreement outlined the following responsibilities of research team and CAB members:

*Kan Lab Member Expectations:*
  • Keep the CAB members informed on results of their feedback/guidance. They will be informed on new research projects and resources developed as a result of their input.
  • Follow up with CAB members to provide them with requested resources and information.
  *CAB Member Expected Duties and Time Commitment:*
  • Four times a year, every CAB member will be expected to attend a quarterly CAB meeting. These meetings will last 1.5 h, and will be scheduled to best accommodate the availability of all members.
  • CAB members will be asked for their advice, opinions, ideas, and concerns on (1) the needs of the community regarding pediatric bladder health and existing knowledge on the topic, (2) how to create the most effective resources to prevent and improve care for pediatric lower urinary tract symptoms, and (3) the degree to which they would like to participate in generating research data and sharing research findings with the community.
  • CAB members will remain on the CAB for a minimum of 1 year, but are invited to remain on the CAB for future years.
*Attendance Policy:*
  • CAB members are expected to attend the quarterly meetings.
  • If a member is unable to attend an upcoming meeting, they will notify Kan Lab members in advance of the meeting.

All CAB members signed the Letter of Agreement prior to the fourth CAB meeting.

#### 3.4.3 Annual report

The annual report was developed, reviewed by the research team, and disseminated via email on December 15, 2023 (Supplementary Data).

#### 3.4.4 Community partner training

*Communities Connecting to Research* training program was completed by all research team members prior to the first CAB meeting and by all CAB members by December 14th, 2023.

### 3.5 CAB evaluation

#### 3.5.1 Post-Meeting survey

There was a 100% response rate for CAB meetings #1, #2, and #4, and a 50% response rate for CAB meeting #3. For CAB meetings #2, #3, and #4, all CAB members in attendance reported that each respective meeting was “very helpful” or “helpful”; for CAB meeting #1, six of seven CAB members in attendance (85.7%) found the meeting “very helpful” while one member (14.3%) found the meeting “neither helpful nor unhelpful”. For CAB meetings #1 and #2, all CAB members felt that 90 min was the “perfect duration”; for CAB meetings #3 and #4, one CAB member in each respective meeting felt that it was “1–30 min too long”. Following the first CAB meeting, three (42.9%) CAB members requested that CAB meeting materials for planned review be provided one-week in advance. This request was met in all future CAB meetings.

#### 3.5.2 Adapted CTSA CAB implementation survey results

All CAB members completed the adapted CTSA CAB Implementation Survey. When asked to identify their responsibilities as a CAB member, all CAB members identified “attend and actively participate in regularly scheduled meetings”, and six (85.7%) identified “disseminate research opportunities and findings in the community” as responsibilities of CAB members (Figure 2A). When asked about methods used to evaluate the effectiveness of the CAB, four CAB members (57.1%) identified “building partnerships”, “number of CAB meetings”, “implementation of CAB recommendations”, and “satisfaction surveys of CAB members” (Figure 2B) as key methods of CAB evaluation. However, one CAB member (14.3%) did not know how the CAB is evaluated.

When asked about the extent that Kan Lab incorporates CAB feedback as an indicator of community impact, four (47.1%) selected “completely” and three (42.9%) selected “a lot” (Figure 2C). When asked about the extent that CAB members are addressing the CAB's purpose and goals as an indicator of community impact, four (57.1%) selected “completely”, two (28.6%) selected “a lot”, and one (14.3%) selected “somewhat” (Figure 2D).

When asked about the greatest challenges to the CAB, five (71.4%) CAB members identified “CAB member attendance” and four (57.1%) identified “CAB members unsure about how to contribute” (Figure 2E). The following were identified as challenges by one CAB member (14.3%), respectively: (1) “meeting logistics (scheduling, location)”, (2) “CAB members don't understand their role”, (3) “communication between meetings”, and (4) “CAB member turnover”.

### 3.6 Bi-directional feedback

**Figure 3:**
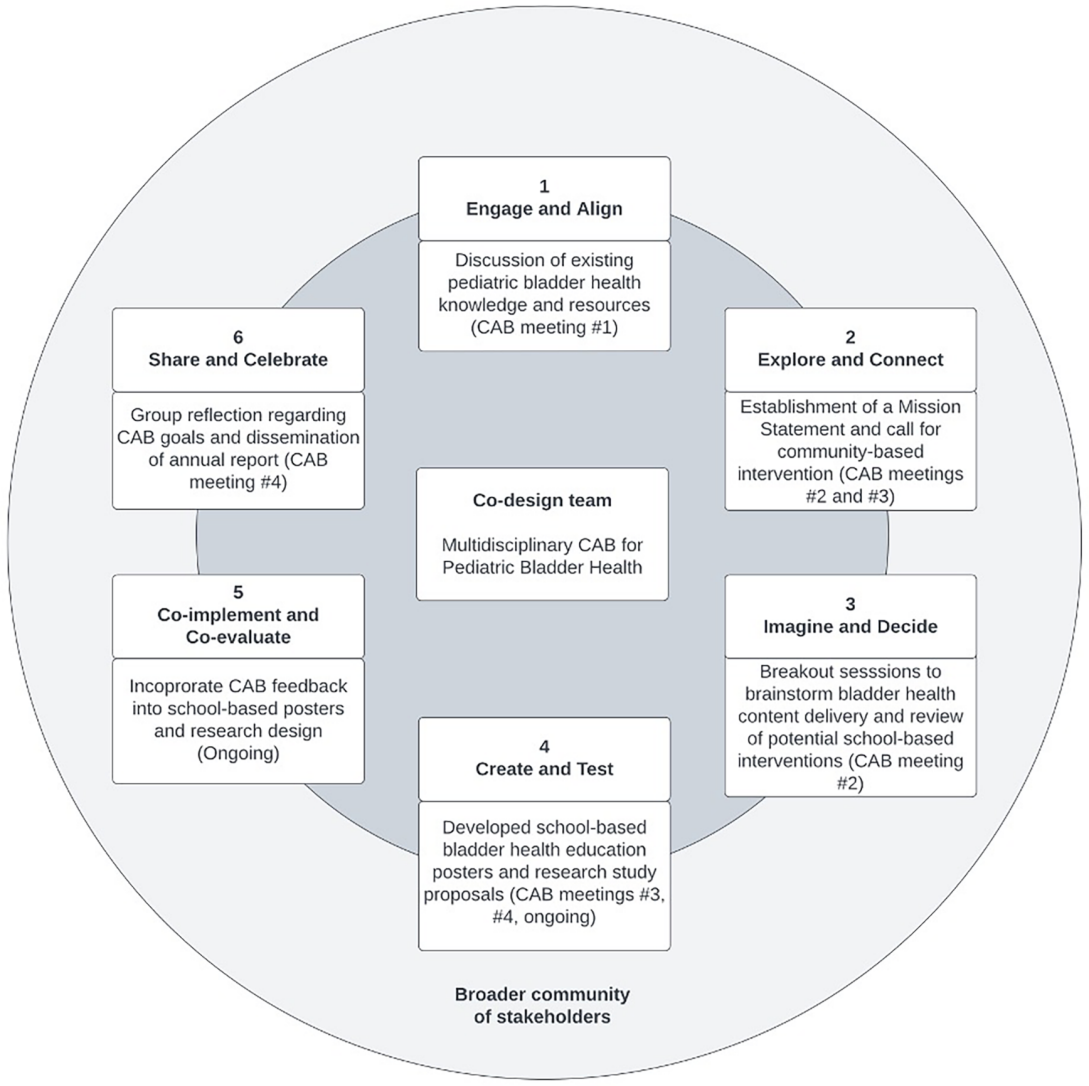
Co-design framework utilized to guide bi-directional feedback. Framework was adapted from Metro North Health (29).

Bi-directional feedback was guided by six stages of the Co-Design framework (Figure 3) (29). Our Co-Design process started with providing introductory information on bladder health and a review of existing bladder health interventions, including education materials, during CAB meeting #1 (engage and align). This facilitated discussions that led to the establishment of the Mission Statement in CAB meetings #2 and #3, as well as a call for (1) bladder health resources to be distributed in different community settings by each member and (2) efforts to address the barriers within a school setting that are obstacles to recommended bladder health behaviors (explore and connect). Breakout sessions in CAB meeting #2 reviewed possible formats to deliver bladder health education resources such as videos, pamphlets, posters, and workshops. The research team also developed and reviewed potential school-based behavioral interventions to assess if they met the expectations for scale and impact (imagine and decide). Following this brainstorming session, we developed ten bladder health posters that could increase awareness of healthy bladder behaviors and presented them for feedback in CAB meeting #3 (create and test). These posters will be modified accordingly and distributed to CAB members in future meetings (co-implement and co-evaluate).

During CAB meeting #4, we engaged in a group reflection to identify areas of improvement and success in accomplishing CAB annual goals. An annual report was created and disseminated to all CAB members (share and celebrate). At the end of four CAB meetings, the group had reached consensus on: (1) the need for the development of tailored resources to distribute to parents, educators, primary care providers, and community members to teach children about healthy bladder behaviors, and (2) barriers to bladder health (e.g., time constraints, low awareness of healthy bladder behaviors), exist within the community, particularly in the school environment. These are illustrated in the meeting takeaways presented in Supplementary Table S3, which inform future directions for Kan Lab research. The varied perspectives offered by CAB members helped inform recommendations for school-based interventions. For example, an educator on the CAB provided insight into the implementation of existing bathroom and water fountain usage rules in the classroom; a parent on the CAB shared their child's perspective on the factors that dictate bathroom cleanliness. Collectively, these perspectives facilitated a broad overview of bladder health in the school environment and allowed us to identify how to improve the effectiveness and impact of our future school-based research projects.

## 4 Discussion

This is the first CAB for pediatric bladder health that brings together community stakeholders and a research team with the common goal of improving pediatric LUTS treatment and prevention. This work demonstrates our group's ability to effectively co-design both a CAB and future research priorities. Here we present how a community-engaged approach helped us in these overall goals.

### 4.1 Lesson 1: CAB members helped to recruit additional members to establish our final group

CAB member recruitment is an iterative process that requires persistent and evolving efforts on the part of research team members. The goal of recruitment is to not only engage individuals with specific experience, but also those who express enthusiasm for the CAB meetings, provide feedback on a range of subjects, and are able to navigate team dynamics in group decision-making. A barrier to consider is that these individuals may not be connected to existing academic recruitment channels. Therefore, we utilized both simple random sampling and non-probability sampling methods including snowball and convenience sampling methods. As recruitment progressed, we began to use non-probability sampling methods more due to unsuccessful recruitment using simple random sampling. For example, no response was received from potential educators or CHWs following our initial random sampling methods. Therefore, we asked CAB members for potential contacts of educators and *El Tímpano* for potential contacts of CHWs for recruitment—both examples of snowball sampling.

These techniques are helpful in settings with a current low awareness of a health concern and therefore, a lack of pre-established community structures to support recruitment. In pediatric bladder health, we see evidence of low awareness of healthy bladder behaviors despite the high prevalence of symptom burden in the community (32, 33). Teachers report low knowledge of healthy bladder behaviors and an absence of formal professional development on the topic, but when educated, play a key role in successful interventions to decrease LUTS in individual students (15). Similarly, parents and pediatricians are familiar with LUTS but unaware of where to access the best resources to support improvement (16, 17). When establishing a CAB, creating, fostering, and leveraging partnerships with the community to engage in non-random sapling is critical to successful recruitment.

To maintain small-group dynamics, we elected to recruit only seven members to our CAB. Consequently, the small number of CAB members may have limited representation in race, gender, and socioeconomic status among the CAB. However, by recruiting individuals who play various roles in their respective communities (i.e., parent, educator, nurse, pediatrician, and/or CHW), we were able to elicit diverse perspectives representative of the broader community responsible for the care and education of children. We are currently conducting additional studies with bilingual and Spanish-speaking families and intend for findings from these studies to expand our CAB to increase inclusivity. Further, we hope to expand our CAB in subsequent years to include additional school staff members and bilingual parents of children with LUTS.

### 4.2 Lesson 2: CAB infrastructure builds trust and generates bi-directional feedback, leading to key insights in our current research program

Co-creation of a Mission Statement, early establishment of expectations and responsibilities, demonstrating respect for members' time, and utilizing member checking approaches to validate our understanding of feedback aided in building trust with our CAB. This foundation fostered open communication throughout meetings #1–4 not just with the research team, but among the members themselves, of whom have different experiences and perspectives to offer. The Co-Design framework supported our shared-decision making process and efforts to translate CAB members' diverse experiences and perspectives into key recommendations for bladder health interventions. This framework has been successfully implemented across disciplines, including development of dental treatment educational materials (34). improvement of a state-wide delirium prevention program (35), and optimization of telehealth programs (36).

The impact of applying this framework to guide effective CAB decision-making is most evident in our work with bladder health content (e.g., school posters). Our qualitative work with local parents and pediatricians had previously provided an important first step in understanding the need for bladder health resources in the community (16, 17). Logistically, conducting additional individual informant interviews to capture both potential solutions and iterative feedback are time consuming and costly for the research program. The CAB's unique format enabled the team to validate our findings from previous qualitative work, provide insight on content format (e.g., posters, pamphlets, etc.), and review first drafts of content over a few hours. The feedback from multiple perspectives helps to optimize the likelihood that our messaging effectively transits to our audience. Our adapted CTSA CAB Implementation Survey results demonstrate that these practices are considered acceptable and impactful to our community. However, one CAB member reported in the survey that CAB members are “somewhat” addressing the CAB's purpose and goals. As this survey was completed anonymously, we do not know the identity of the stakeholder who provided this response, which could be a limitation to our CAB evaluation process. This response highlights the need for further exploration of CAB member experiences in subsequent years of CAB operations. We believe the theory, methods, and deliverables presented in this paper are key to fostering an environment of trust and recommend prioritizing these factors in the establishment of CABs to maximize the positive benefit for both the research team and community members involved.

### 4.3 Lesson 3: the CAB helped to identify a need to shift pediatric LUTS research from the clinic setting to the community setting, with a focus on elementary schools

Utilizing a high degree of interdisciplinary collaboration allowed the CAB to identify key areas of pediatric bladder health to target for improvement in the Northern California Bay Area community. In response to “Bladder Health Questions” proposed in CAB meeting #1 (Supplementary Table S3), each CAB member shared their experience with the practice of healthy bladder behaviors in the community. Consensus was reached among all members that the school environment is a current barrier to healthy bladder behaviors and an opportunity for intervention. CAB members voiced that the elementary school environment can act as a barrier to healthy bladder behaviors due to a lack of available bladder health resources, time to teach healthy bladder behaviors, variation in bathroom utilization privileges, knowledge about healthy bladder behaviors, and cleanliness of bathrooms. These priorities reflect existing literature that demonstrates low awareness of healthy bladder behaviors in parents (17), a lack of publicly available bladder health resources for families and primary care providers (16, 17), unclean bathrooms in school environments (37), and restrictive policies established by teachers who do not promote healthy bladder behaviors (18, 37). Based on our CAB experience, research on pediatric LUTS must shift its focus from the clinic to the community, which represents a paradigm shift in the delivery of LUTS care.

Findings from our CAB provide direction on community-based messaging. Identifying elementary schools as a key setting for future interventions and reviewing bladder health posters aimed at promoting healthy bladder behaviors among students demonstrates (1) community readiness to partner with research teams to improve LUTS care, and (2) the ability for the CAB to continue supporting future research efforts. We will continue to refine future school-based interventions through continuation of our CAB in 2024. Our future research projects aim to improve bathroom access, utilization, and quality in the school environment. CAB members have expressed interest in reviewing materials related to our school-based studies, such as study flyers, study protocols, and/or consent forms that will be shared with parents, students, teachers, and school staff involved in the study. We plan to engage in discussion with CAB members regarding additional involvement such as recruitment, data collection and analysis, and dissemination as our partnership continues.

## 5 Conclusion

This is the first community-engaged approach for pediatric bladder health research that aims to improve delivery of care and can serve as a national example for CE research in the field of pediatric urology. Through bidirectional feedback, we identified a readiness for community-based bladder health resources and identified the school environment as a top priority for intervention. The CAB will continue to play a key role in future community engagement efforts and all aspects of the research process.

## Data Availability

The datasets presented in this article are not readily available in order to protect participant privacy and to keep their responses confidential. This is in line with Stanford IRB’s rules for protection of human subjects. A de-identified dataset can be provided upon request. Requests to access the datasets should be directed to kkan@stanford.edu.
