# Erratum: Creating a community advisory board for pediatric bladder health

**DOI:** 10.3389/fped.2024.1514789

**Published:** 2024-10-30

**Authors:** Frontiers Production Office

**Affiliations:** Frontiers Media SA, Lausanne, Switzerland

**Keywords:** community advisory board, lower urinary tract symptoms, pediatrics, community engagement, urology, community health

An Erratum on Creating a community advisory board for pediatric bladder health By Teehan E, Phord-Toy A, Venkatapuram P and Kan KM (2024). Front. Pediatr. 12: 1396003. doi: 10.3389/fped.2024.1396003

Due to an error, the funder for the article processing charge “Dr. Penniston (Department of Urology, University of Wisconsin-Madison)” was erroneously omitted.

The publisher apologizes for this mistake. The original version of this article has been updated.

