# Supplementary material for: Creating a community advisory board for pediatric bladder health
Source: Front Pediatr. 2024 Jul 16;12:1396003. doi: 10.3389/fped.2024.1396003 (PMC11287218; doi:10.3389/fped.2024.1396003)
Supplement: Supplementary file 1 [file Table1.docx]

**Supplementary Table 1.** Evaluation Metrics for CAB. Various data collection methods were utilized to evaluate the CAB from a multi-dimensional perspective. Format of the table was originally adapted from Fruytier et al.(38)

| **Framework Component** | **Metric** | **Data Collection Method** |
| --- | --- | --- |
| Input | Feeling of preparedness of CAB members | End-of-meeting survey |
|  | CAB meeting priorities | Meeting minutes  Post-meeting survey |
|  | Diversity of CAB members (role within the community and in child care) | Recruitment tracker  Annual report |
|  | Quality and timing of preparation materials | Post-meeting survey |
| Activities/process | Feeling of trust between CAB members | Post-meeting survey |
|  | Attaining expectations of CAB meetings | Post-meeting survey |
|  | Usefulness of CAB meetings | Post-meeting survey  Annual Adapted CTSA Community Advisory Board Implementation survey |
|  | Feelings of transparency | Post-meeting survey |
| Learnings and changes | CAB members understanding of patient’s unmet needs | Annual Adapted CTSA Community Advisory Board Implementation survey  Post-meeting survey |
|  | How meetings informed research goals of Kan Lab | Meeting minutes  Post-meeting survey |
|  | CAB members understanding of patient-relevant outcome measures | Annual Adapted CTSA Community Advisory Board Implementation survey  Post-meeting survey |
|  | CAB members understanding of research decision-making | Annual Adapted CTSA Community Advisory Board Implementation survey  Post-meeting survey |
|  | Planned/recommended actions and changes implemented and not implemented | Meeting minutes  Annual report |
| Short-term impact | Feeling of trust between CAB members and Kan Lab | Annual Adapted CTSA Community Advisory Board Implementation survey  Post-meeting survey |
|  | Expected study participant burden in future research | Developed material review |
|  | Degree to which the CAB demonstrates the value of pediatric LUTS prevention and treatment to the community | Annual Adapted CTSA Community Advisory Board Implementation survey  Annual report |
