# Supplementary material for: Creating a community advisory board for pediatric bladder health
Source: Front Pediatr. 2024 Jul 16;12:1396003. doi: 10.3389/fped.2024.1396003 (PMC11287218; doi:10.3389/fped.2024.1396003)
Supplement: Supplementary file 2 [file Table2.docx]

**Supplementary Table 2.** Questions Included in the Post-Meeting Survey.

| **Areas of Feedback** | **Question** |
| --- | --- |
| Usefulness of CAB meetings | On a scale of 1-5, please rate how helpful you found today’s meeting. |
| Attitudes towards CAB meeting format and duration | On a scale of 1-5, what did you think of the length of today’s meeting? Was it too long, too short, or just right? |
| Overall preparedness of each CAB member prior to meetings | Was there anything not discussed today that you had expected to be discussed? |
| Overall preparedness of each CAB member prior to meetings | Would you like to be provided with anything in advance of the next meeting? If yes, how far in advance of the meeting would you like to be provided the resource? |
| Additional Feedback | Please share any other feedback or suggestions that you may have. |
