# Supplementary material for: Creating a community advisory board for pediatric bladder health
Source: Front Pediatr. 2024 Jul 16;12:1396003. doi: 10.3389/fped.2024.1396003 (PMC11287218; doi:10.3389/fped.2024.1396003)
Supplement: Supplementary file 3 [file Table3.docx]

**Supplementary Table 3.** CAB Quarterly Meetings. The purpose, topics, questions asked to CAB members, main takeaways, and deliverables of each CAB meeting are presented.

| **Date** | **Purpose** | **Topics** | **Questions to the CAB** | **Main Takeaways** | **Meeting Deliverables** |
| --- | --- | --- | --- | --- | --- |
| June 5, 2023 | Welcome/Introduction | 1. Help CAB members gain an understanding of the unique backgrounds of their co-members through the interactive introduction session. 2. Collectively build a set of goals for CAB through level-setting exercises. 3. Obtain feedback on the four bladder health habits. 4. Identify areas of improvement/ gather general feedback for subsequent meetings. | **Introductory Questions:**   1. Why did you decide to join the CAB? 2. What do you hope to take away from the CAB? 3. What comes to mind when you think about pediatric bladder health?   **Bladder Health Questions:**   1. What are your first impressions of the four bladder health habits? 2. What do you think some of the barriers and facilitators are of practicing the four bladder health habits? 3. Who do you think is responsible for making sure children and families know about the four bladder health habits? | - Barriers and facilitators to bladder health exist in the home, school, clinic, and community environments. - Challenges exist with access to clean bathrooms, healthy foods, and reminding children to go to the bathroom outside of the home environment. - Desire from pediatricians, families, teachers, and community members for resources they can use to teach children about good bladder habits. | Reviewed handout outlining overall pediatric bladder health and the four bladder health habits  Received contact for educator who may be interested in joining the CAB |
| September 6, 2023 | Mission statement, CE research training, and bladder health education resource development | 1. Collaborate to finalize CAB Mission Statement. 2. Introduce and discuss shared-decision making process. 3. Obtain feedback on Bladder Basics content (Trailer). 4. Identify stakeholder specific concerns and goals for bladder health program implementation through moderated breakout sessions. 5. Identify areas of improvement/ gather general feedback for subsequent meetings. | **CAB Mission Statement:**   1. Do you think the proposed CAB mission statement encompasses your understanding of the CAB?   **Breakout Sessions:**   1. What content would help you to teach children and families about pediatric bladder health? 2. Would you be interested in videos, worksheets, workshops, school programs, etc.? 3. What audiences would you want to teach about pediatric bladder health?   **Group Discussion:**   1. Does anyone have personal experience that would provide insight into the effectiveness of some of the proposed projects from the breakout rooms? 2. Can anyone connect us with teachers, community organization, or other individuals/groups that would be interested in the following potential projects: 1) School-based interventions, 2) Pediatrician-based programs, 3) Community Health Fairs | - Need tailored content about bladder health for different age groups and settings that do not introduce an additional burden to those who have to teach it. - Proposed using videos, posters, pamphlets, workshops. - Need resources for children, parents, teachers, principals, pediatricians, nurses, and community members. - Need to include prevention of pediatric LUTS in mission statement, in addition to treatment of pediatric LUTS. | Completed 1/3 of CE Research Training  Completed Shared-Decision Making Training and Exercise |
| October 23, 2023 | Letter of agreement, CE research training, bladder health education resource development, adapted CTSA CAB survey | 1. Review Letter of Agreement. 2. Discuss community engaged research (CCR training). 3. Review posters developed from CAB feedback about bladder health. 4. Identify areas of improvement/ gather general feedback for subsequent meetings (CTSA Survey). | 1. Do you think that these posters would capture the attention of kids? 2. Do you think that these posters clearly illustrate the message? 3. How can these posters be improved? 4. Where is the most relevant place to put these posters? | - Bladder posters developed could be very useful for integration into classrooms, bathrooms, gyms, and community centers. - Need simplification of some of the images and make the verbiage used in the posters more specific and tailored to diverse needs of students. - Develop a storyline using the characters shown in the posters to teach bladder health in a fun and memorable way to children. - Consider getting feedback from children about the bladder health posters. | Mission Statement Finalized  Signed Letter of Agreement  Completed Adapted CTSA CAB Implementation Survey  Completed 2/3 of CE Research Training |
| December 4, 2023 | Wrap-Up of CAB Year 1 | 1. Complete community engagement training. 2. Review school-based intervention plan developed from CAB feedback. 3. Identify areas of improvement/ gather general feedback for second year of CAB operations (CTSA CAB Implementation Survey findings). | 1. What are your thoughts/initial reactions to the results of the survey? 2. Is there any aspect of CAB that the questions should have asked but were not included? 3. What has your experience been as a CAB member this year? Please share one takeaway and one piece of advice/ feedback that we can use for next year. | - CAB members felt it was valuable to learn about bladder health from a variety of perspectives offered by different individuals on the CAB. - CAB members expressed that they learned how they can contribute to improving pediatric bladder health in the community and want to be provided with resources to disseminate into the community. - CAB members expressed a desire for in-person meetings during 2024. | Completed CE Research Training |
