# Supplementary material for: Creating a community advisory board for pediatric bladder health
Source: Front Pediatr. 2024 Jul 16;12:1396003. doi: 10.3389/fped.2024.1396003 (PMC11287218; doi:10.3389/fped.2024.1396003)
Supplement: Supplementary file 4 [file Datasheet1.pdf]

Community Advisory Board (CAB) Annual Report 2023

Kan Lab *Thursday, Dec 14, 2023*

This marks the conclusion of the first year of Kan Lab's Community Advisory Board (CAB) for pediatric bladder health. In the first 6 months of 2023, we formed our CAB through recruitment of community members important to the health and learning of children in the San Francisco Bay Area. Our final CAB consisted of two pediatricians, one pediatric registered nurse, two parents, two community health workers, and one educator. In the second half of 2023, we had four CAB meetings, where CAB members and members of the Kan Lab team engaged in insightful conversations about pediatric bladder health in the local community and discussed community needs to improve pediatric bladder health.

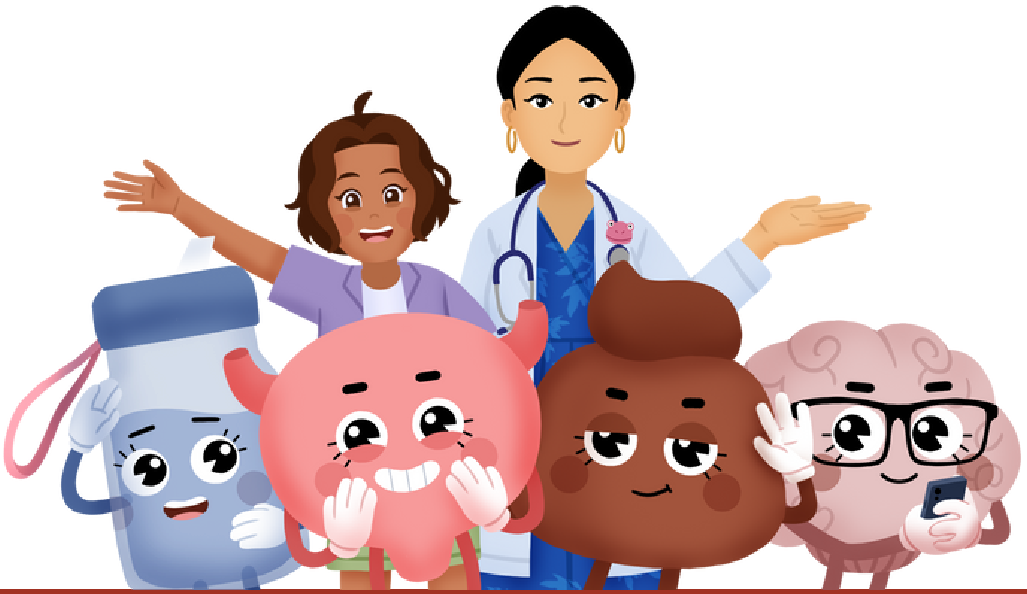

© 2023 Kan Lab and Stanford University. All rights reserved.

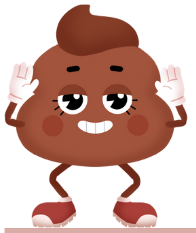

Meeting #1 - June 5th, 2023

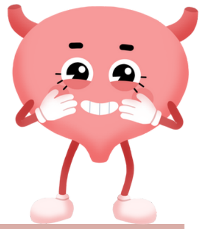

Meeting Objectives

1) Help CAB members gain an understanding of the unique backgrounds of their co-members through the interactive introduction session.
2) Collectively build a set of goals for CAB through level-setting exercises.
3) Obtain feedback on the four bladder health habits.
4) Identify areas of improvement/ gather general feedback for subsequent meetings.

After asking CAB members questions about their current understanding of the CAB and pediatric bladder health, we engaged in discussion about some of the barriers and facilitators to practicing the four good bladder health habits and important individuals who may aid in improving bladder health. We learned that barriers and facilitators to bladder health exist in the home, school, clinic, and community environments. Additionally, we learned that challenges exist with access to clean bathrooms, healthy foods, and reminding children to go to the bathroom outside of the home environment. Finally, there was a desire from pediatricians, families, teachers, and community members for resources they can use to teach children about good bladder habits. As a result, we developed a handout outlining the four bladder health habits. CAB members also provided us with a contact for an educator who was recruited to join the CAB for future meetings.

© 2023 Kan Lab and Stanford University. All rights reserved.

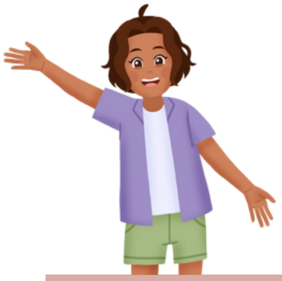

Meeting #2 - September 6th, 2023

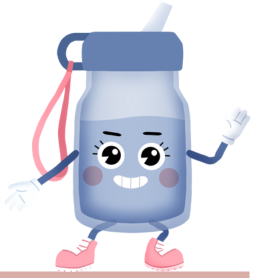

Meeting Objectives

1) Collaborate to finalize CAB Mission Statement.
2) Introduce and discuss shared-decision making process.
3) Obtain feedback on Bladder Basics trailer.
4) Identify stakeholder specific concerns and goals for bladder health program implementation through moderated breakout sessions.
5) identify areas of improvement/ gather general feedback for subsequent meetings.

We first discussed the proposed mission statement of the CAB and received feedback from CAB members to include prevention of pediatric lower urinary tract symptoms (pLUTS) in the mission statement, in addition to the treatment of pLUTS. Next, we went into breakout sessions to discuss what type of content may be helpful to teach children and families about pediatric bladder health, and what audiences may be important to teach. Upon reconvening as a whole CAB, each group shared their discussion points from their breakout session. We learned that there is a need for tailored content about bladder health for different age groups and settings that do not introduce an additional burden to those who have to teach it. CAB members proposed using videos, posters, pamphlets, and workshops to teach children, parents, teachers, principals, pediatricians, nurses, and community members of pediatric bladder health. During this meeting, we also completed 1/3 of the community-engaged research training modules. The training program served to develop a common set of language and understanding of how community-engaged research can be used to induce a tangible impact for community health among CAB members and the Kan Lab team.

© 2023 Kan Lab and Stanford University. All rights reserved.

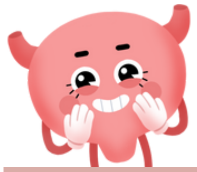

Meeting #3 - October 23rd, 2023

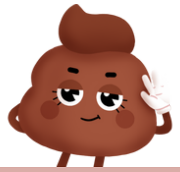

Meeting Objectives

1) Review Letter of Agreement.
2) Discuss community engaged research (CCR training).
3) Review posters developed from CAB feedback about bladder health.
4) Identify areas of improvement/ gather general feedback for subsequent meetings (CTSA Survey).

The meeting began with reviewing the Letter of Agreement, which outlined the responsibilities of CAB members and the Kan Lab team for the 2023 CAB. It also provided the finalized Mission Statement, which incorporated the feedback of CAB members from CAB meeting #2. The finalized Mission Statement is as follows:

> *The purpose of this CAB is to improve care for all families in the Bay Area who are experiencing or are at risk of experiencing pediatric lower urinary tract symptoms. The CAB aims to: 1) Identify common research goals between Kan Lab and CAB stakeholders, 2) elicit ongoing feedback for areas of greatest need within the community, 3) design future solutions that meet community needs effectively by incorporating stakeholder feedback, 4) empower stakeholders to affect change in future projects. Our CAB will value inclusivity, empowerment, and quality care.*

After completing the second third of the community-engaged research training modules, we engaged in discussion about posters developed from CAB feedback about bladder health. We learned that the bladder posters developed could be very useful for integration into classrooms, bathrooms, gyms, and community centers. CAB members also identified that some of the images in the posters need simplification and the verbiage used in the posters should be made to be more specific and tailored to the diverse needs of students. CAB members also suggested developing a storyline using the characters shown in the posters to teach bladder health in a fun and memorable way to children, and to consider getting feedback from children about the bladder health posters. At the conclusion of the meeting, CAB members completed an adapted CTSA CAB implementation survey, to learn about their perspectives and understanding of CAB operations.

© 2023 Kan Lab and Stanford University. All rights reserved.
© 2023 Kan Lab and Stanford University. All rights reserved.

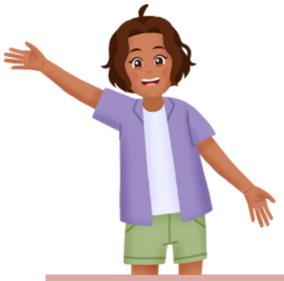

Meeting #4 - December 4th, 2023

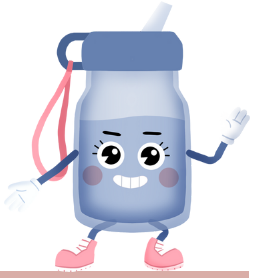

Meeting Objectives

1) Complete community engagement training.
2) Review school-based intervention plan developed from CAB feedback.
3) Identify areas of improvement/ gather general feedback for second year of CAB operations (CTSA CAB Implementation Survey findings).

After completing the community-engaged research training program, we presented CAB members with the results of five of the questions asked on the adapted CTSA CAB implementation survey. The results of the five questions discussed are as follows:

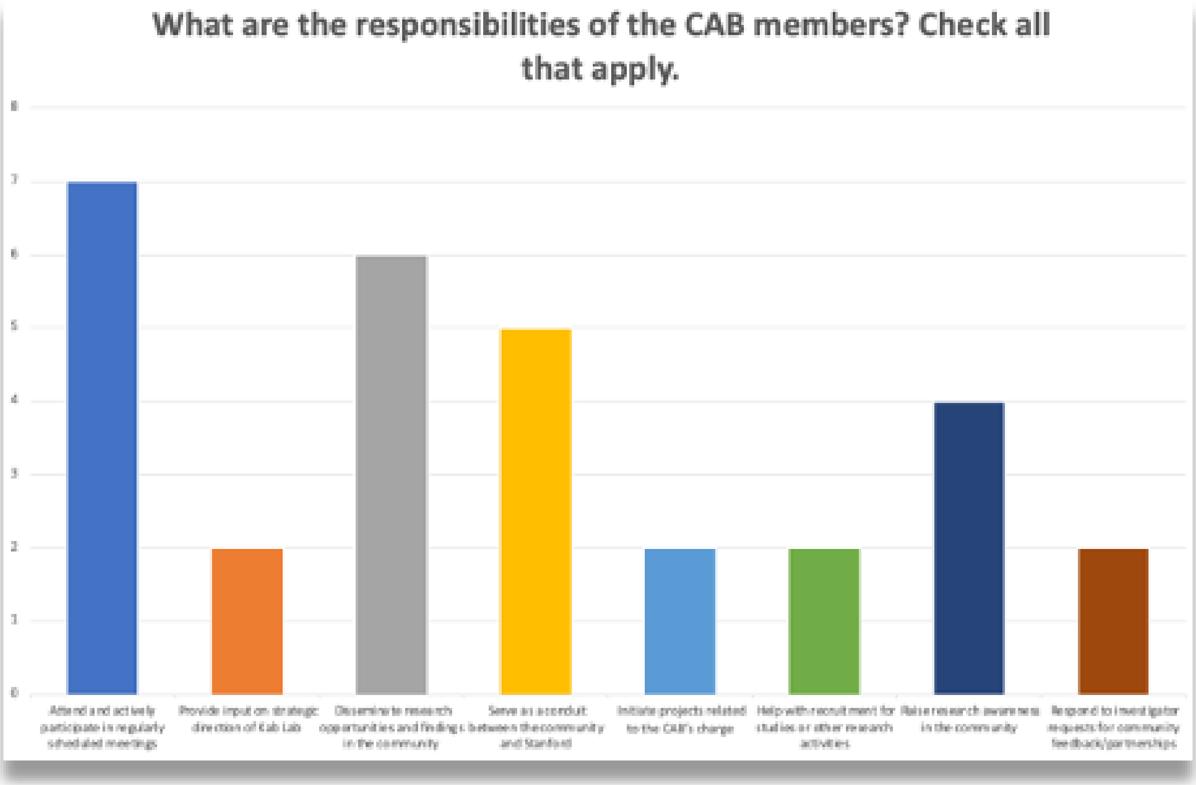

© 2023 Kan Lab and Stanford University. All rights reserved.

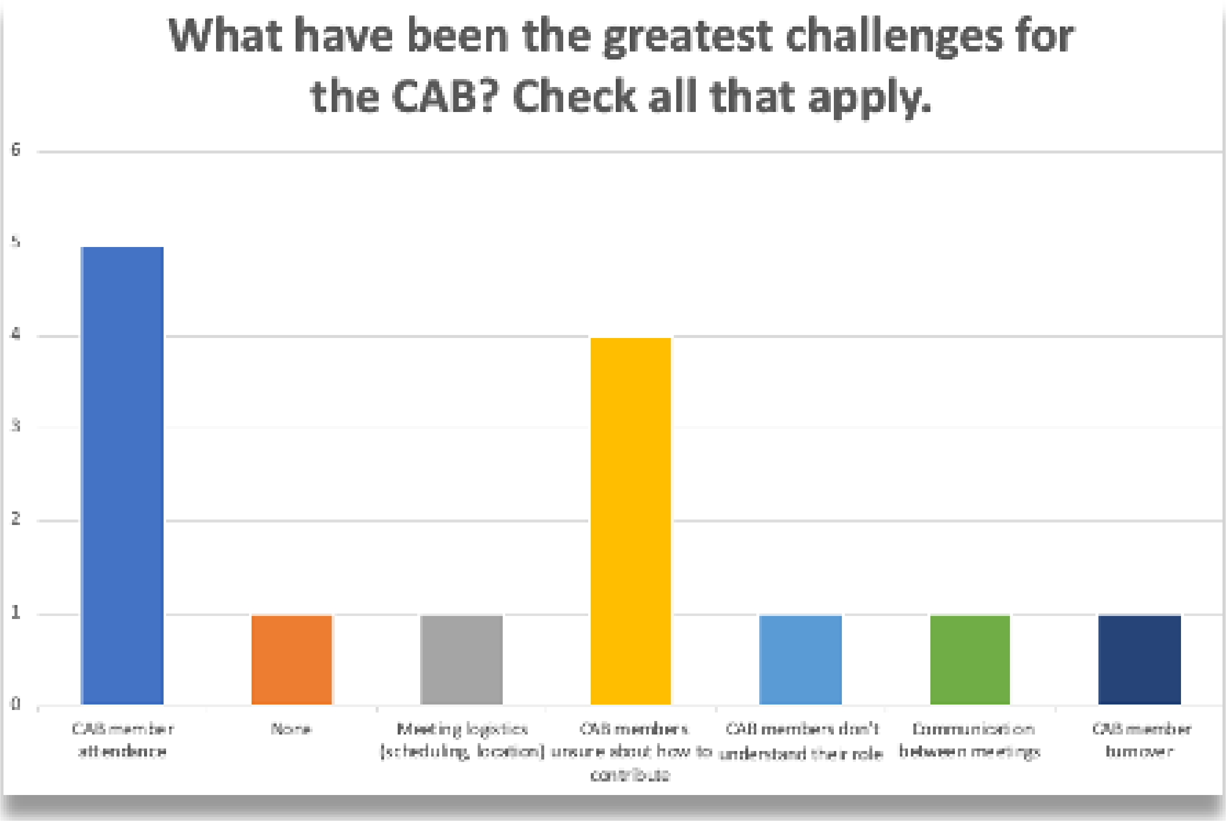

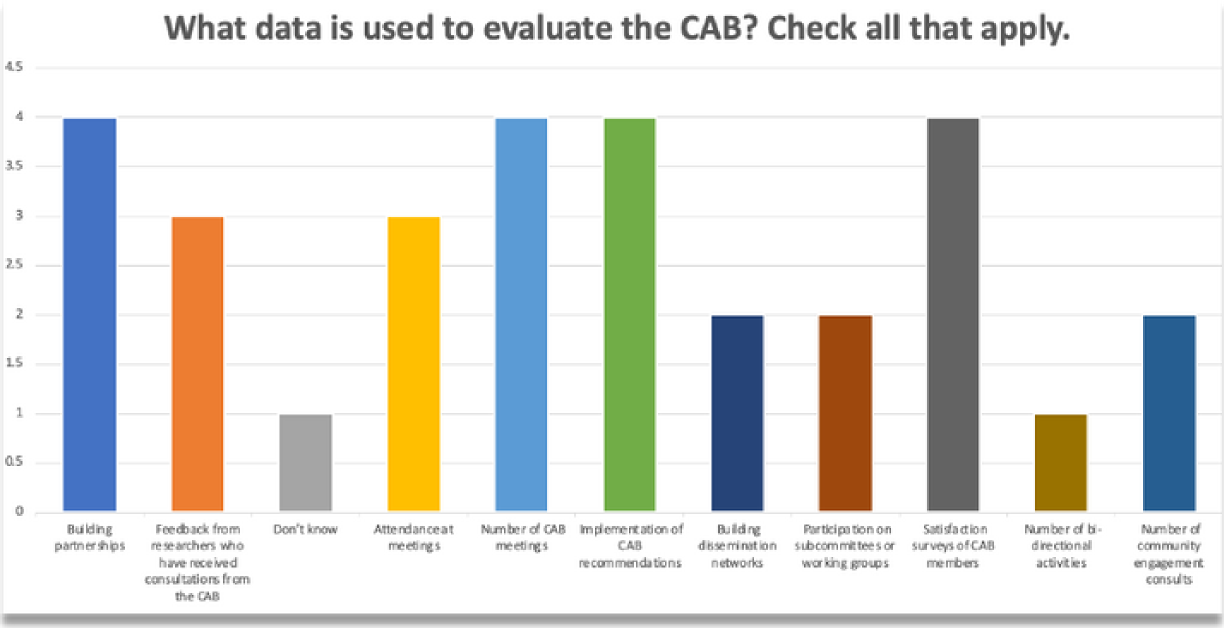

© 2023 Kan Lab and Stanford University. All rights reserved.

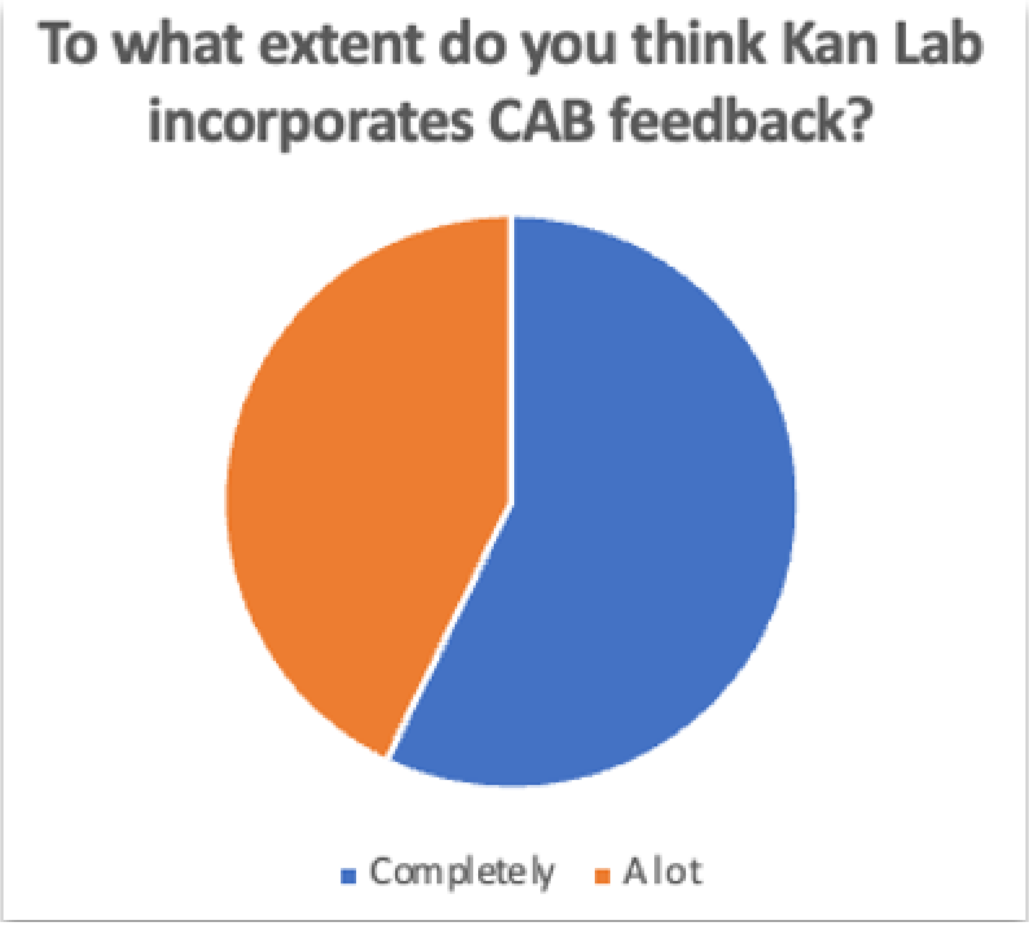

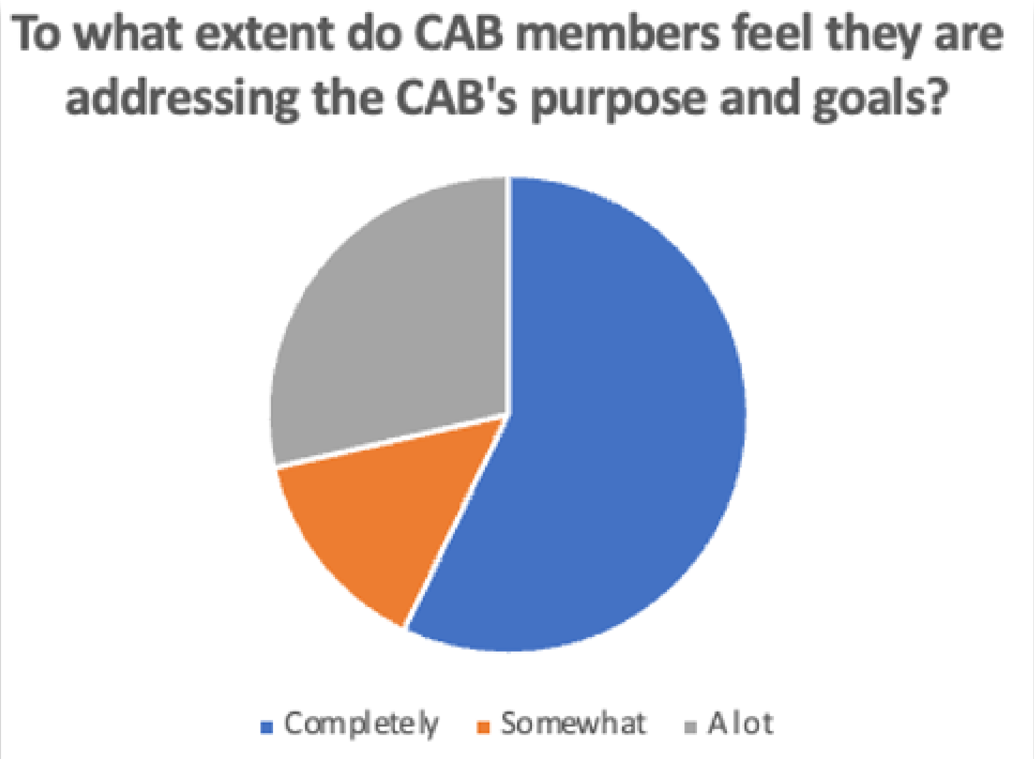

© 2023 Kan Lab and Stanford University. All rights reserved.

Takeaways

CAB members shared that it was valuable to learn about bladder health from a variety of perspectives offered by different individuals on the CAB. They also expressed that they learned how they can contribute to improving pediatric bladder health in the community and want to be provided with resources to disseminate into the community. In discussion about progression of the CAB for 2024, CAB members expressed a desire for in-person meetings, which was a shared sentiment with Kan Lab members. To conclude the meeting, Dr. Kan discussed plans for future school-based research studies that are intended to be heavily discussed with CAB members in 2024 for feedback and guidance on study development and implementation.

At the conclusion of each CAB meeting, an end-of-meeting feedback survey was completed by each CAB member. There was a 100% response rate for CAB meetings #1, #2, and #4. There was a 50% response rate for CAB meeting #3. All CAB members found all four CAB meetings very helpful or helpful, except for the first CAB meeting where six of seven CAB members in attendance (85.7%) found the meeting very helpful. All CAB members felt that 90 minutes was the perfect duration for CAB meetings #1 and #2. For CAB meetings #3 and #4, one CAB member in each respective meeting felt that it was a little too long (1-30 minutes too long). The most common feedback following the first CAB meeting was a request for materials to be reviewed one-week in advance of CAB meetings, of which was fulfilled for all future CAB meetings.

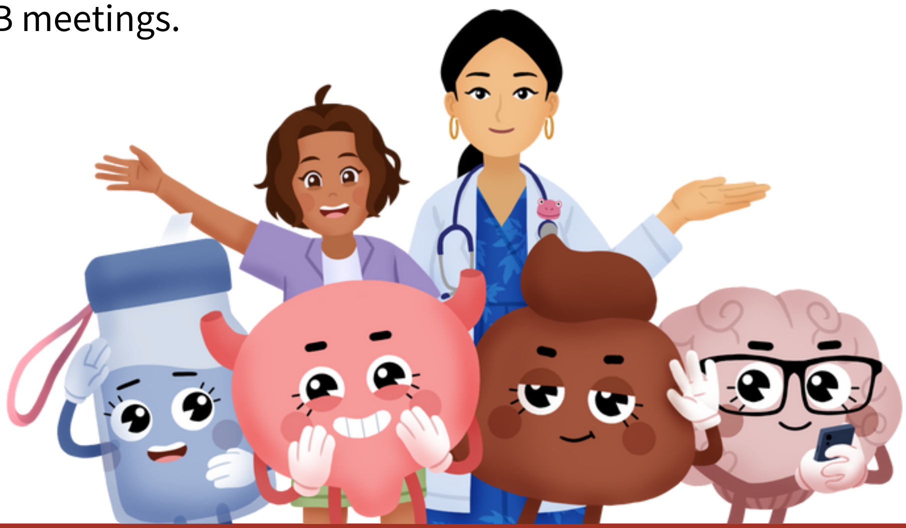

© 2023 Kan Lab and Stanford University. All rights reserved.

What's Next?

We are currently working on a manuscript to publish our findings from our first year of the CAB, in which we share our experiences with CAB recruitment, CAB operations, and CAB evaluation. This will aid in providing guidance to other research teams and physicians about community engagement and the benefits of developing a CAB. Through the manuscript, we hope to encourage more individuals within the scientific community to collaborate with community members to improve the impact of their work.

The first year of Kan Lab's CAB for pediatric bladder health was a success! We want to sincerely thank all CAB members for their participation in the CAB this year. The valuable feedback provided by each CAB member allowed us to identify the needs of the community for pediatric bladder health and provide guidance for the future direction of Kan Lab efforts. We are really excited about future collaboration with CAB members to continue learning about pediatric bladder health in the community.

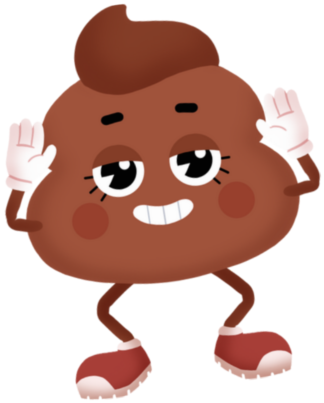

Thank You!

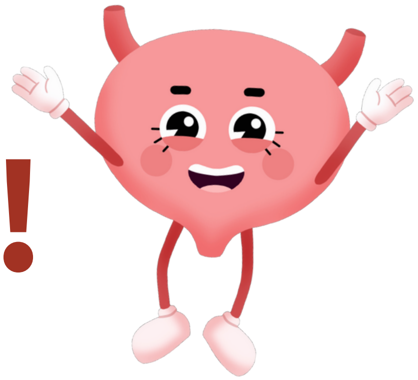

If you have any feedback or questions about our CAB, reach out to:
Dr. Kathleen Kan (kkan@stanford.edu)
Pranaya Venkatapuram (pranayav@stanford.edu)
Emily Teehan (eteehan@stanford.edu)
Ashley Phord-Toy (ashleypt@stanford.edu)

© 2023 Kan Lab and Stanford University. All rights reserved.
