# Supplementary material for: Creating a community advisory board for pediatric bladder health
Source: Front Pediatr. 2024 Jul 16;12:1396003. doi: 10.3389/fped.2024.1396003 (PMC11287218; doi:10.3389/fped.2024.1396003)
Supplement: Supplementary file 5 [file Datasheet2.docx]

**SUPPLEMENTARY REFERENCES**

38. Fruytier SE, Vat LE, Camp R, Houÿez F, De Keyser H, Dunne D, Marchi D, McKeaveney L, Pitt RH, Pittens CACM, et al. Monitoring and Evaluation of Patient Engagement in Health Product Research and Development: Co-Creating a Framework for Community Advisory Boards. *J Patient Cent Res Rev* (2022) 9:46–57. doi: 10.17294/2330-0698.1859
